# The genetic technologies questionnaire: lay judgments about genetic technologies align with ethical theory, are coherent, and predict behaviour

**DOI:** 10.1186/s12910-022-00792-x

**Published:** 2022-05-25

**Authors:** Svenja Küchenhoff, Johannes Doerflinger, Nora Heinzelmann

**Affiliations:** 1 Institute of Neuroscience and Medicine (INM-7: Brain and Behaviour), Research Centre Jülich, Jülich, Germany; 2 Otto Hahn Research Group for Cognitive Neurogenetics, Max Planck Institute for Human Cognitive and Brain Sciences, Leipzig, Germany; 3 Institute of Systems Neuroscience, Heinrich Heine University Düsseldorf, Düsseldorf, Germany; 4 Department of Psychology, University of Konstanz, Konstanz, Germany; 5 Institute of Philosophy, University of Erlangen-Nuremberg, Erlangen, Germany

**Keywords:** Genetic technologies, Genome editing, Applied ethics, Public health ethics, Policymaking, Ethics of technology

## Abstract

**Background:**

Policy regulations of ethically controversial genetic technologies should, on the one hand, be based on ethical principles. On the other hand, they should be socially acceptable to ensure implementation. In addition, they should align with ethical theory. Yet to date we lack a reliable and valid scale to measure the relevant ethical judgements in laypeople. We target this lacuna.

**Methods:**

We developed a scale based on ethical principles to elicit lay judgments: the Genetic Technologies Questionnaire (GTQ). In two pilot studies and a pre-registered main study, we validated the scale in a representative sample of the US population.

**Results:**

The final version of the scale contains 20 items but remains highly reliable even when reduced to five. It also predicts behaviour; for example, ethical judgments as measured by the GTQ predicted hypothetical donations and grocery shopping. In addition, the GTQ may be of interest to policymakers and ethicists because it reveals coherent and ethically justified judgments in laypeople. For instance, the GTQ indicates that ethical judgments are sensitive to possible benefits and harms (in line with utilitarian ethics), but also to ethical principles such as the value of consent-autonomy.

**Conclusions:**

The GTQ can be recommended for research in both experimental psychology and applied ethics, as well as a tool for ethically and empirically informed policymaking.

**Supplementary Information:**

The online version contains supplementary material available at 10.1186/s12910-022-00792-x.

## Background

### Introduction

Genetic technologies have recently been at the heart of much discussion in both academic and non-academic circles [1–3]. This is mainly because their increasingly widespread use has turned ethical issues from thought experiments into live possibilities. For example, the first birth of humans from genetically modified embryos in 2018 prompted a worldwide call by researchers for a moratorium on heritable genome editing [4]. The signatories included Emmanuelle Charpentier who was in 2020 awarded a Nobel Prize for developing the CRISPR/Cas9 genome editing method.

In democracies, ethical judgments of laypeople inform regulations of genetic technologies [5, 6]. However, to even determine those judgments, tools that satisfy the standards of both ethical and empirical research are a prerequisite. However, despite the ongoing debate about the ethics of genetic technologies, to date few studies have addressed both the relevant ethical concerns and abided by methodological standards of psychology, thus meeting the needs of moral philosophers and psychologists alike [7]. In particular, a scale reliably measuring lay judgements about the most ethically controversial issues raised by genetic technologies would aid academic investigation and inform policymakers and regulators.

Our work targets this lacuna. We created and validated a scale to measure ethical judgements about genetic technologies by joining forces from ethics and cognitive sciences. The present paper reports first results from a study in a representative sample. In addition to gaining meaningful insights into laypeople’s ethical views about issues raised by genetic technologies, which align largely with ethical theory, we also find that our scale is predictive of real-world behaviour and differentiates between individual characteristics. In what follows, we first outline the ethical issues arising from genetic technologies (“Methods” section), and then discuss lay judgements about those issues (“Results” section), before presenting the questionnaire to measure those judgements that we developed and validated in a representative sample (“Discussion” section). We discuss the properties of our scale, the relevance of our work for ethics, and its limitations in “Conclusion” section.

### Ethics of genetic technologies

Genetic technologies raise ethical concerns in a vast range of domains such as the moral status of the organisms affected by genetic technologies (e.g., when we ethically assess the use of genetic technologies, should we differentiate between humans and non-human beings?), privacy (e.g., could it be ethically permissible for doctors to share genetic data of patients?), and social justice (e.g., is it fair to allocate public funds for expensive gene therapies?). In what follows, we take these domains in turn.

#### Moral status

Intrusively observing or interfering with nature may be, per se, ethically problematic [8–10]. It may be problematic because it could violate the moral status of the living beings concerned. Moral status is a complex and difficult issue; here, we provide a mere panoramic view of some of its various dimensions. Roughly, the higher the moral status of a being, the more its interests matter morally [11]. Most ethicists agree that having cognitive capacities, having the ability to develop them, or being a member of a species that has them grounds moral status. Relevant cognitive capacities may be consciousness [12], sensitivity to pleasure and pain [13], autonomy [14], or self-awareness [15].

Having the relevant cognitive capacities to a fuller extent or greater degree grounds a higher moral status, other things being equal. For example, ethicists commonly ascribe a lower moral status to living beings that are not or to a lesser degree conscious, sentient, autonomous, or self-aware. In this vein, most animals enjoy a higher moral status than most plants [16], and although this is contested [13, 17], membership of the human species grounds a higher moral status, other things being equal [18, 19]. On this view, using genetic technologies on humans is generally considered as ethically less permissible than using them on non-human living beings like animals, plants, fungi, or bacteria.

The ethical prohibition against interfering with a living being that has moral status may be overruled or outweighed. Depending on the reasons that outweigh or the rights that trump the prohibition to interfere with a being of moral status, it is less or more permissible to violate it. For instance, the reason that your life is threatened and your right to self-defense may arguably trump the prohibition against shooting a tiger; yet your enjoyment of hunting or your desire for a trophy do not.

In the context of genetic technology, a strong reason overriding the prohibition to interfere with a being that has moral status is a person’s consent. Consent is, roughly, the voluntary agreement given by a fully informed and decision-capacitated agent [20]. In most cases, if a person consents to an interference, the ethical prohibition against it is overruled. If the living being in question cannot consent, it remains pro tanto ethically impermissible to interfere. This could be the case when the being is not yet or temporarily not able to consent because, say, they are asleep or an embryo [21].

The ethical permissibility to interfere also varies with the severity or nature of the interference. For example, taking photos of a living being with moral status is, ceteris paribus, less severe than killing or torturing it. Accordingly, the latter is less permissible than the former. The same is true for genetic technologies: the more profoundly they interfere with a living being, the less ethically permissible it is to do so, other things being equal. For one thing, genetic testing is a less severe interference than genetic editing, and thus the former is more ethically permissible than the latter.

In sum, to determine the degree to which it is ethically permissible, if at all, to use genetic technologies in a given case, it is necessary to consider the moral status of the living beings affected by it, the potential benefits or reasons promoted by it, and the severity of the interference. Approaches that take several or all of these considerations into account often employ cost–benefit analyses or a weighing of moral reasons or goods. For example, from a utilitarian perspective, different options of various dimensions are commensurable with respect to the potential benefits and harms they bring about [13, 22, 24]. On this view, whether and to what degree it is ethically permissible to use genetic technologies on a living being ultimately reduces to the question of whether doing so would promote overall well-being.

#### Data privacy

That privacy is ethically valuable has been noted at least since Aristotle ([23], I), who distinguished the public sphere (“polis”) from the private one (“oikos”; cf. [24–27]). Because of its ethical value, privacy is generally regarded as being protected by moral rights, and violating it is regarded as pro tanto ethically impermissible [28]. Nowadays, privacy typically concerns property and dwelling, communication, or personal data.

Genetic technologies raise ethical concerns about privacy, and in particular about the privacy of genetic data. Collecting or sharing a human being’s genetic data is pro tanto ethically impermissible [29–32]. Genetic data is ethically sensitive for at least two reasons. On the one hand, individuals can typically be uniquely identified based on their genetic data. On the other hand, access to genetic data is usually difficult to control; e.g., we can hardly prohibit access to the genetic data that we leave behind in hairs or dead cells wherever we go. In these respects, genetic data may differ from other sensitive personal data from, say, telecommunications. Ethical judgments about genetic technologies should thus be sensitive to the value of privacy of genetic data and the ethical obligations and prohibitions that protect them.

#### Social justice

Although definitions of “justice” vary greatly, many capture the core idea that justice consists in rendering each their due (“suum cuique”; [33]). Roughly, then, justice concerns the impartial rights or claims of individual agents. More specifically, social justice in a modern liberal society is often understood as a set of principles, practices, and institutions agents would hypothetically agree on in advance [34–36]. For example, it could be argued that in a just society, inequality may be ethically permissible only if it is attached to offices and positions that are equally accessible to all members or benefit the least-advantaged members [35].

Because just principles, practices, and institutions include regulations and usage of genetic technologies, the ethics of genetic technologies is in part concerned with issues of social justice. For example, principles of social justice require that, ceteris paribus, everyone have fair and equal access to public goods like genetic technologies, or that individuals are treated fairly and justly in light of different genetic profiles.

### Laypeople’s moral judgments of genetic technologies

If laypeople’s moral judgments align with the ethical theory as outlined in the previous section, empirical evidence can be expected to support the following hypotheses (preregistered on the Open Science Framework, OSF^1^). First, laypeople will judge the use of genetic technologies as morally worse when applied to humans compared to nonhumans. This difference would reflect the difference in the moral status of the affected organisms. Second, genetic technologies will be judged as morally worse when they interfere more severely with the being concerned. Specifically, genome editing will be judged as morally worse than testing, other things being equal. Third, because human adults can consent to the use of genetic technologies but embryos cannot, laypeople will judge their usage in embryos as morally worse than their usage in adults. Fourth, laypeople’s ethical judgments could align with a utilitarian cost–benefit analysis. For example, when genetic technology is used for a greater benefit (e.g., to prevent a fatal disease or cancer or to fight world poverty), it will be judged as morally better than when employed for a smaller benefit (e.g., to prevent a disease that is perceived as less severe, or to improve the taste of foods).

In previous surveys it was observed that respondents from the US [3], China [37], and a worldwide sample [38] generally rate genetic technologies favourably if these technologies are used for therapeutic purposes. In addition, in a study where attitudes about genetic research were assessed [39], respondents also indicated a favourable view. Therefore, we predicted that lay people would generally be in favour of genetic technologies.

Past research has also identified relationships between ethical judgments and demographic variables, knowledge, and personality. We therefore also preregistered the following hypotheses. More educated [7, 38], wealthier [38, 39], less religious [40], and more liberal participants [37, 39] would rate genetic technologies as morally better, other things being equal. Moreover, ethical approval of genetic technologies would be greater when participants know more about those technologies [3, 41]. For example, people well versed in genetic technologies will understand the different implications embryonic vs. adult gene editing will have on the body and if it will or will not affect future offspring. Relatedly, expecting that a finding for genetically modified foods generalises to genetic technologies, more extreme ethical judgments would align with greater presumed knowledge [41]. Participants were also expected to rate genetic technologies as morally better if they had prior exposure to genetic testing [38]. Lastly, we hypothesised that individuals would rate genetic technologies as ethically better the less they considered purity and sanctity as important moral values [9] and the more they saw themselves as open to experience [42].

## Methods

### Questionnaire construction

We developed the Genetic Technologies Questionnaire (GTQ) to assess the sensitivity of laypeople’s moral judgments to the moral status of the living being affected, data privacy concerns, and issues of social justice. As one clearly distinguishable feature of moral status, we chose membership of the human or a non-human species. As another salient feature, we categorically varied the severity of the interference with a being that has moral status: genetic testing versus genome editing. Thus, we developed questionnaire items covering six domains: genetic testing of humans, genetic testing of non-humans, genome editing of humans, genome editing of non-humans, data privacy, and social justice. All items described, as far as possible, real-life applications of genetic technologies. For example, they concerned the actual use of genetic technologies on crops or farm animals, in reproductive medicine, or in scientific research.

We also used language and topics that laypersons could understand without further information and explanation. Items and response options were formulated in a neutral way to avoid framing effects or other unwanted influences of wording—an issue that [7] criticized in a review of previous questionnaire research on laypeople’s evaluation of genetic technologies.

Moreover, we were interested in ethical judgements about genetic technologies specifically, not about technology more generally or incidental features of contexts in which genetic technologies are used. Therefore, we employed a contrastive design (described in further detail below).

We refined the questionnaire in two consecutive pilot studies and validated it in a representative sample of the US population. The aim of the pilot studies was to generate a set of candidate items, explore the relevant themes in laypeople’s moral judgment of genetic technologies, and select the most suitable items. While the literature in applied ethics guided item generation, we were not committed to preserving the six domains outlined above in the final version of the scale, let alone establishing sub-scales. Because genetic technologies are complex, we expected that judgments about them would be sensitive to a multitude of different aspects. Our goal was to develop one coherent general scale for ethical judgments about genetic technologies that would be behaviourally relevant.

We employed a contrastive design following a well-established tradition in experimental philosophy [43] and bioethics [44, 45]. That is, as all our items were intended to measure ethical judgments about genetic technologies, we developed, for each of them, a nearly identical item that replaced expressions about genetic technologies with expressions about conventional technologies. Thus, for example, one item of our questionnaire read:

Changing the genome of farm animals in order to improve their wellbeing is….

Participants completed the claim by choosing a response ranging from “morally bad” to “morally good” on a six-point Likert scale. The corresponding contrast item read:

Changing the hormones of farm animals in order to improve their wellbeing is….

Comparing responses to target and contrast item allows to attribute differences in ethical judgments more straightforwardly to the difference between genetic versus conventional technologies, rather than to, say, ethical judgments about farming or animal wellbeing. In addition to the GTQ, we thus also used a matched Conventional Technologies Questionnaire (CTQ). The Ethics Committee of the University of Konstanz approved all studies (approval no. 33/2018). Materials and data are available on the OSF.^2^

#### Pilot Study 1: Judging genetic and conventional technologies

We administered a questionnaire with candidate items for the GTQ as part of a more extensive survey of teenagers attending an outreach event at the German Cancer Research Centre, which uses genetic technologies to develop new cancer treatments. The study was explained in detail to the teenage participants, and they and their parents gave informed consent to participation. We also randomly assigned participants at an interdisciplinary summer school for Ph.D. students and postdocs to fill out a questionnaire with the GTQ or CTQ candidate items. To increase statistical power, we pooled the data from both samples. Fifty-two participants from the outreach event and 70 participants from the summer school returned complete questionnaires, resulting in a total sample size of 122 (66 female, one did not indicate gender; age: *M* = 27.0, *SD* = 12.0, range 12–68).

We used a contrastive design with two 35-item versions of the questionnaire (the GTQ and the CTQ). The items were statements about the ethical permissibility of using genetic or conventional technologies in various contexts. The response format was a five-point scale ranging from *strongly disagree* (1) to *strongly agree* (6). All items were coded so that high values indicate a positive attitude towards the respective technologies.

We found that the mean ethical judgment for the target items from the GTQ (*M* = 3.47, *SD* = 0.54) was significantly higher than that of the contrast items from the CTQ (*M* = 3.25, *SD* = 0.40), *t*(76.9) = 2.42, *p* = 0.018. However, the group that filled out the CTQ differed from the one that filled out the GTQ, as the latter but not the former included the entire teenager sample. Therefore, we also calculated a mixed linear model with the questionnaire type as fixed effect and participants and items as random effects. This yielded a significant effect of questionnaire type, i.e., participants judged genetic technologies as ethically more permissible than conventional technologies, *β* = 0.32, *SE*_*β*_ = 0.11, *t*(120) = 2.97, *p* = 0.003), even when variation between participants was accounted for in the model. However, when age was entered as a fixed effect in addition to the questionnaire type into a mixed model controlling for participant and item, the effect of questionnaire type was no longer significant, *β* = 0.18, *SE*_*β*_ = 0.12, *t*(119) = 1.47, *p* = 0.143. Instead, age was a significant predictor, *β* = − 0.01, *SE*_*β*_ < 0.01, *t*(120) = 2.30, *p* = 0.022.

Before assembling the next version of our questionnaire, we interviewed six participants and asked them to verbally respond to and “think aloud” about our items, as recommended in [46, 47]. This allowed us to identify issues of wording and connotation that may have led to confusion or led respondents astray. Finally, based on the results from our pilot study and the interviews, we refined and selected the next set of items for the GTQ and CTQ.

#### Pilot study 2: Item selection

We administered the CTQ or the GTQ to 30 participants on the online platform Prolific. On average, participants took about 16 min to complete either of the questionnaires. They were compensated with £3. Each questionnaire consisted of 48 items, eight items for each of the six ethical dimensions. We adjusted the wording of our question, asking participants to complete claims such as “Genetic testing of crops to improve them for farming is …” by selecting an answer on a six-point Likert scale ranging from “morally good” (1) to “morally bad” (6).

To compare whether our contrastive design detected differences in ethical judgments of genetic versus conventional questionnaires, we calculated the mean rating of the CTQ (*M* = 3.15) and the GTQ (*M* = 3.70), *t*(24.4) = − 2.66, *p* = 0.014. To account for interindividual differences, we also calculated a mixed linear model that predicted the ethical judgment by group and controlled for random effects of participants and items. This yielded a significant effect of questionnaire type, *β* = 0.67, *t*(28.0) = 3.33, *p* = 0.002, indicating genetic technologies were seen as ethically worse than conventional technologies, over and above differences between participants and items.

Cronbach’s alpha for the 48 items of the GTQ was = 0.86, suggesting that the questionnaire had sufficient reliability. To select the items for the final 30-item version of the GTQ, we used the following three criteria. The first was content: each moral dimension identified from the ethical literature should be represented in the questionnaire. Therefore, we included at least one item referring to genome editing of humans and nonhumans, genetic testing of humans and nonhumans, data privacy, and social justice, in the questionnaire. A second criterion was to generate an internally highly consistent scale, therefore we selected items that correlated highly with the mean of the other items. Our third criterion was the difference to contrast items. As we aimed for a questionnaire specific for genetic technologies, the GTQ should be judged differently from the CTQ. We thus selected target items that differed from their contrast item counterpart. Overall, the best 30 items according to these three measures make up our final version of the questionnaire (GTQ30). The corresponding contrast items make up the conventional technologies questionnaire (CTQ30).

### Validation study

We validated the final version of the 30-items GTQ (Table 3) in a representative sample of the US population on the online platform Prolific. We measured the internal consistency of the GTQ30, tested the relations of ethical judgments as measured by the GTQ to demographic variables and individual differences (Big Five Openness and Moral Foundations Purity), and assessed the GTQ’s predictive validity with respect to social financial decisions. Furthermore, we used a contrastive design to compare the moral judgments of genetic (GTQ30) and conventional technologies (CTQ30).

We hypothesised that the internal consistency of the scale would be 0.85 or higher, as measured by Crohnbach’s alpha, and 0.80 or higher when reduced to 20 items; that ethical approval of genetic technologies would correlate positively with openness to experience, income, education, knowledge and liberalism, and negatively with purity. We also expected that lay judgements would be sensitive to ethical values like the moral status of the living being affected by a genetic technology, data privacy, and social justice. In addition, we hypothesised that individuals would preferentially treat others with moral values similar to their own, as revealed by assignments in a third-person dictator-game. We predicted that GTQ score would correlate with hypothetical donations—individuals who judged genetic technologies as morally better should allocate larger sums to organisations endorsing genetic technologies—and self-reports of behaviour—individuals who viewed genetic technologies favourably should be more prone to buy genetically modified foods. Our pre-registered hypotheses are listed in Table 1 and, together with all data and materials, on the OSF.^3^,^4^

**Table 1:** Results for hypotheses preregistered on the open science framework

*Questionnaire reliability and shared variance*	
The internal consistency of the scale is 0.85 or higher	✔
When reduced to the 20 items that correlate highest with the overall-score, the internal consistency of the scale will be 0.8 or higher	✔
Participants rate genetic technologies as morally good (above the midpoint of the scale)	✔
The rated moral goodness of genetic technologies is, on average, higher than that of conventional technologies	✘
The Genetic Technologies Questionnaire (GTQ) explains more variance of the respondents' choices in a third party dictator game in which money is distributed between an individual in favour of genetic technologies and an individual opposed to genetic technologies than the Conventional Technologies Questionnaire (CTQ)	✘
The GTQ explains more variance of the respondents' hypothetical donation choices towards charities who support genetic technologies than the CTQ	✘
The GTQ explains more variance of the respondents' self-reported purchases of genetically modified food than the CTQ	✔
*Validity and predictivity*	
The higher participants score for Openness to Experience, the better they rate genetic technologies (mean rating GTQ, and Item GEN1)	✘
The higher participants score for Purity/Sanctity in the Moral Foundations Questionnaire, the worse they rate genetic technologies (mean rating GTQ, and Item GEN1)	✘
Participants distribute more money in a third-party dictator game to other participants who share their view of genetic technologies (predicted by the mean rating of the GTQ, and Item GEN1)	✘
Participants assign a greater share of donations in a hypothetical case to a charity that is aligned with their views on genetic technologies than to one that is not (based on the mean rating of the GTQ, and Item GEN1)	✔
Endorsement of genetic technologies measured by the GTQ is a significant predictor of consumer behavior. Particularly buying genetically modified food	✔
The higher participants' household income, the better they rate genetic technologies (mean rating GTQ, and Item GEN1)	✔
The higher the participant’s education (measure of education level, years of education) the higher the mean rating GTQ, and item GEN1	✔
The more religious participants consider themselves to be, the worse they rate genome editing (GT15-30)	✘
The more liberal participants consider themselves to be, the better they rate genetic technologies (mean rating GTQ, and item GEN1)	✔
Participants who voted for the Republican candidate rate genetic technologies lower than participants who voted for the Democratic candidate (mean rating GTQ, and item GEN1)	✘
Participants who already had experience with genetic tests rate genetic technologies as morally better (mean rating GTQ, and item GEN1)	✘
The more participants think they know about genetic technologies, the more extreme (trending away from the midpoint of the scale) they rate the morality of genetic technologies (positive or negative)	✘
Objective knowledge about genetics is negatively correlated with opposition to genetic technologies	✔
A discrepancy between self-assessed and objective knowledge about genetic technologies is positively correlated with opposition to genetic technologies	✘
*Predictive power of single items/differences in content within the questionnaire*	
Genetic editing of human adults is regarded as better than that of embryos (the mean rating of GT18 is greater than that of GT19, that of GT15 is greater than that of GT22)	✔
Overall, ratings of genetic testing (GT1-8) correlate with ratings of genome editing (items GT15-30)	✔
Overall, ratings of genome editing are lower (morally worse) than of genetic testing (the mean rating of GT15-30 is lower than that of GT1-8)	✔
Participants self-identified as male rate the use of genetic technologies on animals (items GT5, 6, 23, 24, 27, 29) as morally better than participants self-identified as female	?
Participants rate genetic technologies as morally better when they are used to improve nutritional value (GT28) or fight world poverty (GT25) than to improve taste (GT26), and when they are used to improve wellbeing (GT23) rather than to increase efficiency (GT6)	✔ ✘
Genome editing of embryos is rated as morally better when performed in order to prevent a fatal disease (GT17) than when used to prevent influenza (GT19)	✔
Genome editing of human adults is rated as morally better when performed in order to treat cancer (GT20) than when used to protect them against influenza (GT18)	✘

✔ statistically significant evidence, ✘ no statistically significant evidence, ✔✘: evidence for some items, ?: not tested

#### Participants and design

As Prolific requires at least 300 participants for a representative sample, we chose *N* = 300 as our sample size and used the representative sample option on the platform. The participants were randomly assigned to the target (GTQ30) and the contrast (CTQ30) conditions. Thus, the expected sample size of participants responding to the GTQ was 150. The statistical power to detect a sufficient internal consistency (Cronbach’s *α* = 0.80) with this sample compared to the null hypothesis that the internal consistency would be lower (Cronbach’s *α* = 0.70) was 1 − *β* = 0.95, calculated as described by [48]. Furthermore, a sensitivity analysis with G*power [49] for the contrastive comparison of the GTQ and the CTQ showed that a sample of 300 participants was large enough to detect small to medium-sized effects (*d* = 0.29) with a probability of 1 − *β* = 0.80.

We collected data from 300 participants (149 female, three did not indicate gender; age: *M* = 46.3, *SD* = 15.8, range 19–78). They were representative of the US population regarding age, sex, and ethnicity, based on the US Census Bureau (see Table 2). On average, participants took 15 min to complete the study and were paid £2.35 and a bonus ranging from £0 to £1 based on another participant’s decision in the study.

**Table 2:** Participant sample (N = 300), validation study, compared to US Census Data

	Numbers	Percentage	US census 2018
*Ethnicity*			
White	192	.64	.78
Black	40	.13	.13
Asian	25	.08	.06
Mixed	15	.05	0.3 for other and mixed ethnicities
Other	10	.03	
N/A	18	.06	
*Sex*			
Male	137	.46	.48
Female	145	.48	.52
N/A	18	.06	
*Age*			
18–27	37	.12	.17
28–37	65	.22	.18
38–47	44	.15	.16
48–58	41	.14	.17
58 +	94	.31	.32
N/A	19	.06	

Census data taken from https://www.census.gov/programs-surveys/cps/data.html, accessed in July 2021

#### Procedure

Participants were randomly assigned to one of two groups. One group first completed the GTQ30, the other group responded to the CTQ30. Both groups provided ratings of moral goodness or badness on a 6-point Likert scale ranging from (1) “morally bad” to (6) “morally good,” and the remainder of the experiment was identical. Next, as a single-item measure of their general attitude towards genetic technologies, we asked participants to complete the claim “Genetic technologies are…” using a 6-point Likert scale ranging from (1) “morally bad” to (6) “morally good.”

This was followed by a self-assessment and test of participants’ knowledge about genetic technologies. We probed self-assessment by asking, “Compared to others, how much do you know about genetic technologies?” and recorded responses on a 7-point Likert scale ranging from “much less than others” to “much more than others.” Participants then completed a short test of their genetic knowledge, taken from [41], which prompted them to assess claims like “It is the father’s genes that decide whether the baby is a boy or a girl.”

To probe possible relations with personality, we administered the 10-item short version of the Big Five Inventory [50]. Furthermore, as the literature suggests links between the moral foundation of Purity and Sanctity and attitude towards technology [9, 10], we administered the Purity/Sanctity items of the Moral Foundations Questionnaire [51].

Next, we assessed three relevant measures to evaluate the predictive validity of the GTQ. The first was a third-party dictator game. We asked participants to split a £1 bonus payment between two other participants of the study who had rated genetic technologies as morally good or morally bad, respectively. The only given information about the recipients of the bonus was their moral judgment concerning genetic technologies. After the study, the participants were grouped based on their GTQ score, and for each participant, one other participant with a high GTQ score and one other participant with a low GTQ score were randomly drawn to receive the allotted bonus. Second, we measured donation preferences. Participants made hypothetical donations to two charities on two occasions. On the first occasion, they allocated money between two charities that promoted the development of either genetically modified or conventionally grown crops to fight malnutrition and hunger in developing countries. The other allocation was between two charities that advocated either genetic or conventional screening tests for individuals suffering from severe diseases. For both allocation questions, the participants were asked how they would split £100 between the two charities on a visual analogue slider coded from 0 (£0) to 100 (£100). The allocation decisions were significantly correlated, *r* = 0.32, *p* < 0.001, and averaged into a single scale. Third, we asked participants how often they had bought genetically modified food in the last 6 months on a five-point scale from “never” to “always.”

We also gauged religiosity and political orientation to detect possible correlations with ethical judgments about genetic technologies. We measured religiosity with two items: We asked participants to indicate how religious they were on a five-point scale ranging from “not at all religious” to “very religious,” and how often they attended religious services on a five-point scale ranging from “not at all” to “daily.” Responses to the two items were highly correlated (*r* = 0.75), and the items were averaged into a single scale. We also asked two questions about political orientation. On the one hand, we asked participants to indicate their political orientation on a seven-point scale from “extremely liberal” to “extremely conservative.” On the other hand, we asked them how they voted in the last presidential election (“Democrat,” “Republican,” “other,” or “did not vote/I don’t remember”).

To explore whether personal experience might affect ethical judgments about genetic technologies (cf. [52]), we also asked participants whether they or any of their close friends or relatives ever had a genetic test performed (“yes” or “no”).

As measures of socio-economic status, we determined participants’ education and household income. We measured education with two items: level of education and years of education. Participants indicated their level of education on eight levels (e.g., “less than high school degree,” “doctoral degree,” “professional degree, JD, MD”) and the number of years of education they had completed. We measured annual household income in seven increments from below $15 k to over $100 k. These measures were significantly correlated (*r*s > 0.27). We *z*-standardized the SES items and averaged them into a single social status scale. To test the pre-registrated hypotheses, we also used the individual SES items separately.

In line with Prolific policies, we included a catch item to detect participants who paid insufficient attention to instructions and questions. Responses of four participants who failed the catch item were excluded, and Prolific recruited four matched participants in their place who answered the catch items correctly.

## Results

The GTQ reliably measured subjects’ ethical judgments about genetic technologies, was related to participant demographics and knowledge, showed good construct validity in predicting (real, hypothetical, and self-reported) behaviour, and revealed meaningful ethical judgments. We report these results in turn (see Table 1 for a list of results for all preregistered hypotheses).

### Reliability

The overall internal consistency of the 30-items scale (GTQ30) as measured by Cronbach’s alpha was *α* = 0.95, *CI* [0.94–0.96], which indicates that the items of our questionnaire reliably measure the same construct, viz. ethical judgments about genetic technologies. As some researchers might be interested in a shorter questionnaire, we also calculated the internal consistency of the 20 items with the highest total correlation with the overall score (GTQ20); this resulted in *α* = 0.95, *CI* [0.94–0.96]. Finally, a scale consisting of the top five items (GTQ5) still had an acceptable internal consistency, *α* = 0.89, *CI* [0.87–0.92]. Table 3 provides an overview of all items. Note that the GTQ5 only covers the topics of genome editing in humans and nonhumans. All analyses reported below concerning the GTQ-score are conducted using the GTQ30.

**Table 3:** Items in the GTQ and their corresponding contrast items

GTQ	CTQ
Genetic testing to determine the risk of Down’s syndrome for an embryo in utero is…	Ultrasound scans to determine the risk of Down’s syndrome for an embryo in utero are…
Prescribing genetic tests for healthy women in order to identify markers for breast cancer is …	Prescribing x-ray tests for healthy women in order to identify early stages of breast cancer is …
Using genetic tests to determine if one carries markers for hereditary diseases before deciding to conceive a child is …	Investigating one’s family history for signs of hereditary diseases before deciding to conceive a child is …
Performing genetic tests on consenting adult humans for medical research is …	Performing clinical tests on consenting adult humans for medical research is …
Conducting harmless genetic tests on animals for scientific research is…	Conducting harmless behavioural tests on animals for scientific research is…
*Optimising the breeding of farm animals through genetic testing is…*	Optimising the breeding of farm animals through clinical testing is…
*Performing invasive genetic tests on wild plants to monitor and conserve ecosystems is…*	Performing invasive biochemical tests on wild plants to monitor and conserve ecosystems is…
*Genetic testing of crops to improve them for farming is…*	Selective breeding of crops to improve them for farming is …
Consider a patient with a hereditary disease who has a sibling with similar genes. For the doctor, informing the sibling of the patient’s disease despite privacy concerns is…	Consider a patient who develops a disease from malnutrition and has a partner with similar eating habits. For the doctor, informing the partner of the patient’s disease despite privacy concerns is…
Supporting genetic testing despite privacy concerns is…	Supporting data retention in telecommunication despite privacy concerns is…
For insurers, requesting genetic tests from healthy adults in order to assess their health risks is …	For insurers, requesting medical screenings from healthy adults in order to assess their health risks is …
*Using public health funds on expensive gene therapies is …*	Using public health funds on expensive chemotherapies is …
Taking into account the genetic profile of applicants with respect to genetic diseases when hiring a kindergarten teacher is…	Taking into account the medical history of applicants when hiring a kindergarten teacher is…
Mitigating a criminal sentence due to the offender’s genetic predisposition is …	Mitigating a criminal sentence due to the offender's problematic childhood is …
*Using genome editing on consenting adults to enhance their cognitive performance is …*	Using neurochemical substances on consenting adults to enhance their cognitive performance is …
*Changing the genomes of human embryos for medical research without destroying them is…*	Changing the cell membranes of human embryos for medical research without destroying them is…
*Changing the genomes of human embryos to ensure they will not develop a fatal disease is …*	Using pre-implantation diagnostics on human embryos to ensure they will not develop a fatal disease is …
***Genome editing of human adults to protect them against influenza is …***	Vaccinating human adults to protect them against influenza is …
***Changing the genome of human embryos to ensure they will not get influenza is …***	Using vaccination on human embryos to ensure they will not get influenza is …
*Using risky genome editing therapies for the medical treatment of cancer patients is …*	Using risky chemotherapies for the medical treatment of cancer patients is …
*Testing for the risk of genome editing on consenting adults is …*	Testing for the risk of new chemotherapies on consenting adults is …
*Using genome editing to enhance the cognitive development of human embryos in underprivileged families is …*	Using medical drugs to enhance the cognitive development of human embryos in underprivileged families is …
***Changing the genome of farm animals in order to improve their wellbeing is …***	Changing the hormones of farm animals in order to improve their wellbeing is …
***Editing the genome of farm animals to reduce costs without harming them is …***	Changing the hormone balance of farm animals to reduce costs without harming them is …
*Editing the genome of crops in order to fight world poverty is …*	Using fertilisers and pesticides on crops in order to fight world poverty is …
*Editing the genome of foods to improve their taste is …*	Adding artificial flavors to foods to improve their taste is …
*Editing the genome of animals to make it possible for animal organs to be transplanted to humans is …*	Selectively breeding animals in order to make it possible for animal organs to be transplanted to humans is …
*Editing the genome of crops to improve their nutritional value is…*	Cross-breeding crops to improve their nutritional value is…
*Editing the genome of wild animals to make them immune against certain diseases is …*	Vaccinating wild animals to make them immune against certain diseases is …
***Editing the genome of plants to improve crops for farming is…***	Selectively breeding plants to improve crops for farming is…

Answers were given on a 6-point Likert scale ranging from (1) “morally bad” to (6) “morally good”. Italicized items are included in the GTQ20, bold items are included in the GTQ5

### Demographics, personality, and prior knowledge

Table 4 provides an overview of the correlations of the GTQ30 score and individual difference variables. The correlation between the GTQ score and political orientation was not significant (*p* > 0.05). Nonetheless, a trend suggests that conservative participants tend to judge genetic technologies as morally less permissible, *r* = − 0.13, *p* = 0.118. The correlation between the GTQ and SES was significant and positive, *r* = 0.25, *p* = 0.002. The SES indicator is a composite of income and education. As the correlation of the GTQ with education (*r* = 0.23, *p* = 0.005) is stronger than with income (*r* = 0.16, *p* = 0.054), education seems to be the primary driver of the SES effect. An exploratory follow-up regression analysis predicting the GTQ-score based on both income and education found that education was a significant predictor, *β* = 0.20, *SE*_*β*_ = 0.09, *t*(142) = 2.31, *p* = 0.023, while income was not, *β* = 0.05, *SE*_*β*_ = 0.04, *t*(142) = 1.10, *p* = 0.272. A nonsignificant trend suggested that female participants tended to rate genetic technologies as morally less permissible (*M* = 3.61, *SD* = 0.93) than male participants (*M* = 3.88, *SD* = 1.00), *t*(128.74) = − 1.69, *p* = 0.093. In addition, age was negatively related to the GTQ rating of moral permissibility, *r* = − 0.28, *p* < 0.001. For an item by item summary of correlations with demographic variables see the Additional file 1.

**Table 4:** Correlations between the GTQ score and individual difference variables

	1	2	3	4	5	6	7	8	9	10	11	12
(1) GTQ		− .13	.25^*^	.16^+^	.23^*^	.17^*^	.13	− .07	− .09	− .08	− .28^**^	− .14^+^
(2) Political orientation			.08	.19^*^	.00	− .21^*^	− .17^*^	− .18^*^	.42^***^	.39^***^	.28^***^	.23^*^
(3) SES				.67^***^	.92^***^	.33^***^	.27^**^	− .06	.13	.19^*^	.04	− .19^*^
(4) Income					.33^***^	.19^*^	.21^*^	− .10	.19^*^	.15^+^	− .05	− .26^*^
(5) Education						.32^***^	.23^**^	− .03	.06	.17^*^	.08	− .11
(6) Knowledge tested							.26^**^	.12	− .21^**^	− .16^+^	− .10	.05
(7) Knowledge subjective								.10	− .08	− .08	− .19^*^	− .16^+^
(8) Big-5 openness									− .21^**^	− .17^*^	.03	.12
(9) MFQ Purity										.62^***^	.29^**^	− .07
(10) Religiosity											.29	− .13
(11) Age												.02
(12) Sex (0 = male, 1 = female)												

Pearson correlations, level of significance, two-sided test against 0: +  = .10, * = .05, ** = .01, *** < .001

We also analysed whether presumed and actual knowledge about genetic technologies was related to bioethical judgments about those technologies. That is, we asked participants to report how much they knew about genetic technologies, and we tested their actual knowledge in this domain. While participants’ presumed knowledge was no significant predictor of the GTQ score (*p* = 0.133), their actual knowledge was: the more participants actually knew about genetic technologies, the better they rated them morally, *β* = 0.17, *p* = 0.036.

We found no statistically significant evidence for links between bioethical judgments in the GTQ and openness to experience (*p* = 0.418), purity values on the moral foundation questionnaire (*p* = 0.264), prior personal exposure to genetic testing (*p* = 0.387), or religiosity (*p* = 0.360).

### Predictive validity

The GTQ score was highly correlated with participants’ general endorsement of genetic technologies asked in a single item, *r* = 0.77, *t*(145) = 14.63, *p* < 0.001. To detect possible effects of participants’ ethical judgments on hypothetical donation decisions, we performed a linear regression, with the share of donations participants allocated on the hypothetical allocation questions to charities supporting the use of genetic technologies as the dependent variable and the GTQ score as the predictor. Morally favourable views of genetic technologies on the GTQ were related to a higher share of hypothetical donations given to those charities in favour of genetic technologies, *β* = 6.94, *SE*_*β*_ = 1.56, *t*(145) = 4.44, *p* < 0.001. In the third-party dictator game, the higher the participants’ GTQ score, the more money they allocated descriptively to another participant who was also in favour of genetic technologies, although this trend was not statistically significant, *β* = 1.89, *SE*_*β*_ = 1.58, *t*(145) = 1.20, *p* = 0.233.

To detect a possible relationship between participants’ ethical judgments and their reported shopping behaviour, we set up a regression model that predicted the frequency of buying genetically modified food by the mean GTQ score. We found that the better participants judged genetic technologies on average, the more often they reportedly bought genetically modified food, *β* = 0.38, *SE*_*β*_ = 0.08, *t*(140.8) = 4.56, *p* < 0.001.

### Moral judgments

Examining bioethical judgments, we first performed a sanity check and determined whether ethical judgments about genetic testing (items GT1 to GT8 of the GTQ30) correlated positively with ethical judgments about genome editing (items GT15 to GT30). We found a strong correlation between the two, *r* = 0.77, *t*(145) = 14.46, *p* < 0.001. Still, participants regarded genetic testing (*M* = 4.41, *SD* = 0.98) as morally better than genome editing (*M* = 3.78, *SD* = 1.19), *t*(281.4) = 4.92, *p* < 0.001.

Overall, the participants who filled out the GTQ rated genetic technologies as morally good (*M* = 3.72, *SD* = 0.98, scale midpoint = 3.5, 6 = good), but ratings were worse than those of conventional technologies as rated by participants who filled out the CTQ (*M* = 4.07, *SD* = 0.75), *t*(273.1) = 3.47, *p* < 0.001. To test for this difference while also accounting for by-subject and by-item variability, we calculated a mixed linear model and compared the mean ethical judgments of our target questionnaire with those of the contrastive questionnaire. The CTQ group had significantly higher scores than the GTQ group, *β* = 0.35, *SE*_*β*_ = 0.10, *t*(298) = 3.48, *p* < 0.001.

Ethical judgments varied with the moral status of the being on and the purpose for which genetic technologies were deployed. For instance, participants judged genome editing in human adults (*M* = 3.85, *SD* = 1.47) as better than in human embryos (*M* = 3.19, *SD* = 1.51), *t*(291.8) = 3.85, *p* < 0.001.

Participants also judged genetic technologies as morally better when used to fight world poverty or improve the nutritional value of foods rather than their taste. More precisely, they rated genetic technologies used to improve nutritional value (*M* = 4.39, *SD* = 1.52) as morally better than genetic technologies used to improve taste (*M* = 3.60, *SD* = 1.65), *t*(290) = 4.26, *p* < 0.001. They also rated using genetic technologies against poverty (*M* = 4.68, *SD* = 1.51) as morally better than using them to improve taste, *t*(290) = 4.26, *p* < 0.001. Genome editing was rated as morally better when used to prevent a fatal disease (*M* = 3.94, *SD* = 1.65) rather than influenza, which need not be fatal (*M* = 3.44, *SD* = 1.73), *t*(292) = 2.56, *p* = 0.011.

Ethical judgments also reflected concerns about data privacy and social justice. As an example of the former, participants rated a doctor’s sharing of genetic data as morally worse (*M* = 3.12, *SD* = 1.28) than the sharing of nutrition data (*M* = 3.60, *SD* = 1.01), *t*(298.0) = 2.58, *p* = 0.012. As an example of the latter, participants rated the use of public health funds for gene therapies as morally worse (*M* = 3.27, *SD* = 1.58) than their use for chemotherapies (*M* = 4.40, *SD* = 1.34), *t*(285.9) = 6.72, *p* < 0.001.

## Discussion

We developed a scale that is both suited as a tool for experimental research and as a measure of judgments about ethically relevant issues identified from the philosophical literature: the GTQ30. In addition, we offer a 20-item version of this questionnaire (GTQ20), which is also highly reliable, covers the ethical domains homogeneously, and may thus be of interest for both experimentalists and moral philosophers. Finally, we also developed a matched contrast questionnaire about conventional technologies (CTQ). These questionnaires are available in Table 3 and on our OSF website alongside all our other materials, data, and scripts.^5^

### Scale properties

Our scale was highly reliable in a representative sample of the US population, correlated meaningfully with individual characteristics, and showed good construct validity in predicting behaviour. In line with prior research, we found that ethical ratings about genetic technologies as measured by our scale correlated positively with income [38, 39], education [7, 38], and liberal political orientation [37, 39]. Moreover, ethical approval of genetic technologies was greater when participants knew more about those technologies, aligning with previous findings [3, 41].

A novel result is that we find bioethical judgments to correlate with behavioural decision-making: measures on our scale predicted allocations in a dictator game, self-reported shopping behaviour, and hypothetical donations. Note that only the third-party dictator game, in which we measured if like-minded participants would be treated favourably, measured real-life behaviour, and that all other decisions concerned self-reported and hypothetical behaviour. These results indicate that our scale may measure ethical judgments that align with social discourse and actions. Consequently, it could prove a valuable tool to predict behaviour in experimental studies on the one hand and to gauge support for and implementation of policy regulations on the other hand.

Overall, we found that participants rated genetic technologies as morally good, thereby replicating previous findings from the literature [3, 7, 37–39]. However, our contrastive design allows for a finer-grained interpretation: we also find that participants rated conventional technologies as morally even better than genetic technologies. This may suggest that, although participants have favourable moral opinions of technologies generally, they are more critical of some technologies than of others (here of genetic as compared to conventional technologies). Future work may put this finding even further into perspective by comparing technologies with regard to their domain of application (e.g., medical, agricultural, research) or their method (e.g., genetic, neural, AI). Researchers interested in the full range of ethical issues may wish to rely on the 30-item version (GTQ30) whilst those who need a more economic version might utilise the GTQ20 or GTQ5 instead which are still reliable but do not cover all potentially relevant aspects. Researchers interested in genetic technologies only can use the GTQ on its own; those who wish to compare it to other kinds of technologies may rely on the combination of GTQ and CTQ.

### Relevance for ethics

The questionnaire and our study sheds light on two strands of research in ethics. First, our scale elicits laypeople’s judgments about core ethical issues arising from genetic technologies, which pertain to moral status, data privacy, and social justice. Second, we find ethical judgments as measured by our scale to be coherent and sound. Experimental and empirically-informed philosophers have long worried that lay and expert judgements, as well as real-life behaviour, do not conform to norms of morality, rationality, and epistemology [53–57]. Our findings do not raise these worries, at least not for judgements of genetic technologies and when measured with our scale and design. On the contrary, we find that ethical judgments are coherent, abide by norms of ethical theory, and align with behaviour.

More specifically, we found that ethical judgments were sensitive to the moral status of the beings on which they were deployed and the severity of the reasons for which the moral status of those beings was compromised, thus converging with ethical theory. For instance, it is pro tanto ethically impermissible to interfere with beings that are not yet or temporarily unable to give their informed consent; this is in accordance with our finding that participants judged genome editing in human adults as better than in human embryos, in line with previous research [2].

Moreover, participants rated genetic technologies as morally better when used to fight world poverty or to improve the nutritional value of foods rather than their taste. Genome editing was rated morally better when used to prevent a fatal disease rather than influenza, which may but need not be fatal. These findings align with cost–benefit analyses in applied ethics and converge with utilitarian theory more generally. That is, participants seemed to trade off potential risks of genetic technologies against their potential benefits. They also distinguished prudential goods like an improved taste from altruistic or moral goods such as prosocial benefits like improved healthcare, rating the latter as morally better.

Participants also seemed to take issues of data privacy and social justice into account in their ethical judgments. For instance, in their view, it was morally worse for a doctor to share a patient’s genetic disposition rather than their nutrition with a third party, which aligns with philosophical views that genetic data is particularly ethically sensitive [29–32]. Similarly, participants regarded social justice issues as morally worse when arising from genetic technologies than conventional technologies. For example, they found the allocation of public goods and services based on genetic profile and predisposition morally problematic and, interestingly, significantly worse than allocation based on socioeconomic background or health profile information. Not only do these views align with general philosophical accounts of social justice [35, 36], they also extend them to the particular social justice issues arising from genetic technologies.

### Limitations

The work presented in this paper is limited in at least two ways. First, we did not find empirical support for some of our preregistered hypotheses and thus failed to replicate some findings reported in previous research. In particular, we did not find significant evidence for hypothesised correlations of bioethical judgments and individual moral values (as measured by the Moral Foundations Questionnaire), openness to experience (as measured by a subset of Big Five items), religiosity (as measured by self-assessment and reported frequency of attending religious services), and prior exposure to genetic testing. We had expected that individuals would rate genetic technologies as ethically better the less they considered purity and sanctity as important moral values [9], the more they saw themselves as open to experience [42], the less religious they rated themselves [40], and the less exposure they had had to genetic testing [38]. We found no evidence for any of these hypotheses. There may be several reasons for these null results, such as that any possible effect was much smaller than anticipated or that these individual characteristics do not affect bioethical judgments straightforwardly. Clearly, future research examining these possibilities will inform our understanding of the connections between an individual’s personality and their judgements about specific ethical issues. Further studies may also examine more complex lay judgements about, say, ethical dilemmas, and relate them to the GTQ scores.

A second limitation of our paper is that it only reports main results from a representative sample of the US population, which may or may not generalise to other cultures and countries. Bioethical judgments may be susceptible to idiosyncratic views of particular cultures or policy regulations in different legislations. For example, survey respondents from the European economic area (EEA) have been found to be more opposed to the use of genetic technology in adults and embryos than those from the US [2], and while genetically modified crops are common in the US, they are not in the EU, which effectively banned them from 1999 to 2004. Future studies may address these shortcomings by comparing bioethical judgments across cultures on the one hand and by testing for correlational and causal links between culture or legislation and bioethical judgement on the other.

## Conclusion

Overall, our work may provide experimentalists with a reliable and valid tool to quantify ethical judgments about genetic technologies. Moreover, it may be of interest to ethicists and moral psychologists in that it presents judgments by a representative sample of the US population on the full range of ethical concerns about genetic technologies. Finally, not only do we hope that our scale and findings will further foster interdisciplinary research, but we also aspire to contribute to public discourse and policymaking concerned with genetic technologies in particular and ethical issues in general.

## Supplementary Information

**Additional file 1:** Supplemental Material.

## Data Availability

The datasets generated and/or analysed during the current study are available in the Open Science Framework (OSF) repository, https://osf.io/nmpxu/?view_only=c247714486cf47ac96698b168cc18dbe

## Footnotes

1 https://osf.io/fjhtx/?view_only=971e70cd09da48e58fee68b3daf7118f.

2 https://osf.io/nmpxu/?view_only=c247714486cf47ac96698b168cc18dbe.

3 https://osf.io/fjhtx/?view_only=971e70cd09da48e58fee68b3daf7118f.

4 https://osf.io/nmpxu/?view_only=c247714486cf47ac96698b168cc18dbe.

5 https://osf.io/nmpxu/?view_only=c247714486cf47ac96698b168cc18dbe.
