# Supplementary material for: The genetic technologies questionnaire: lay judgments about genetic technologies align with ethical theory, are coherent, and predict behaviour
Source: BMC Med Ethics. 2022 May 25;23:54. doi: 10.1186/s12910-022-00792-x (PMC9134650; doi:10.1186/s12910-022-00792-x)
Supplement: Supplementary file 1 — Additional file 1: Supplemental Material. [file 12910_2022_792_MOESM1_ESM.docx]

**Table S1**

Correlations of demographic variables and items of the GTQ

|  | Politics^a^ | Test^b^ | Income^c^ | Age^d^ | Sex^e^ | Religious^f^ | Education^g^ |
| --- | --- | --- | --- | --- | --- | --- | --- |
| GT1 | -.04 | -.06 | .18* | -.15 | -.07 | -.06 | .12 |
| GT2 | -.11 | -.04 | .11 | -.09 | .05 | -.17* | .18* |
| GT3 | -.17* | -.07 | .11 | -.05 | .09 | -.17* | .07 |
| GT4 | -.13 | -.12 | .03 | -.14 | .12 | -.08 | .11 |
| GT5 | .10 | -.07 | .10 | -.13 | -.16 | .00 | .15 |
| GT6 | .04 | -.14 | .16 | .01 | -.14 | .06 | .27** |
| GT7 | -.04 | -.17* | .14 | -.10 | -.07 | -.12 | .22** |
| GT8 | -.09 | -.08 | .14 | -.19* | -.03 | -.12 | .21** |
| GT9 | .10 | .04 | .20* | -.09 | -.21* | .08 | .2* |
| GT10 | .11 | -.01 | .18* | -.10 | -.16 | .00 | .14 |
| GT11 | .12 | .15 | .10 | -.17* | -.06 | .21** | .01 |
| GT12 | -.09 | -.09 | .12 | -.21* | -.14 | -.02 | .19* |
| GT13 | .09 | .04 | .09 | -.19* | -.23** | .21** | -.10 |
| GT14 | -.07 | .04 | .02 | -.11 | -.05 | .21* | .10 |
| GT15 | -.15 | .03 | .08 | -.19* | -.02 | -.03 | .09 |
| GT16 | -.19* | -.04 | .04 | -.39*** | -.03 | -.06 | .13 |
| GT17 | -.18* | -.02 | .07 | -.26** | -.13 | -.18* | .14 |
| GT18 | -.19* | .07 | .08 | -.26** | -.09 | -.10 | .12 |
| GT19 | -.13 | -.01 | .12 | -.35*** | -.1 | -.07 | .07 |
| GT20 | .03 | -.05 | .03 | .02 | -.13 | -.03 | .23** |
| GT21 | -.12 | -.08 | .01 | -.11 | -.1 | -.19* | .10 |
| GT22 | -.10 | .12 | -.03 | -.32*** | -.1 | -.04 | .00 |
| GT23 | -.13 | -.14 | .09 | -.26** | -.13 | -.04 | .13 |
| GT24 | -.01 | -.16* | .15 | -.17 | -.16 | .08 | .16* |
| GT25 | -.11 | -.09 | .14 | -.24** | -.02 | -.06 | .21* |
| GT26 | -.12 | -.06 | .12 | -.21* | -.02 | -.09 | .14 |
| GT27 | -.05 | -.07 | .16 | -.17* | -.09 | -.04 | .28*** |
| GT28 | -.10 | -.04 | .13 | -.18* | .01 | -.04 | .23** |
| GT29 | -.16* | .07 | .11 | -.25** | -.01 | -.12 | .13 |
| GT30 | -.12 | -.05 | .08 | -.13 | -.02 | -.07 | .18* |

*Note.* Statistical significance, two-sided test against *r* = 0: * *p* < .05; ** *p* < .01; *** *p* < .001. ^a^ Political orientation: 1 = extremely liberal, 7 = extremely conservative; ^b^ Have you or any of your close friends/relatives ever had a genetic test performed?: 1 = yes, 2 = no; ^c^ Yearly household income in brackets: 1 = less than $15,000, 2 = $15,000 - $24,999; 3 = $25,000 - $34,999, 4 = $35,000 - $49,999, 5 = $50,000 - $74,999, 6 = $75,000 - $100,000, 7 = more than $100,000; ^d^ Age in years; ^e^ Sex: 0 = male, 1 = female; ^f^ mean of normalized religiosity items; ^g^ mean of normalized education items.

**Table S2**

Items in the GTQ

| Item Number | Item |
| --- | --- |
| GT1 | Genetic testing to determine the risk of Down’s syndrome for an embryo in utero is... |
| GT2 | Prescribing genetic tests for healthy women in order to identify markers for breast cancer is ... |
| GT3 | Using genetic tests to determine if one carries markers for hereditary diseases before deciding to conceive a child is ... |
| GT4 | Performing genetic tests on consenting adult humans for medical research is ... |
| GT5 | Conducting harmless genetic tests on animals for scientific research is... |
| GT6 | Optimising the breeding of farm animals through genetic testing is... |
| GT7 | Performing invasive genetic tests on wild plants to monitor and conserve ecosystems is... |
| GT8 | Genetic testing of crops to improve them for farming is... |
| GT9 | Consider a patient with a hereditary disease who has a sibling with similar genes. For the doctor, informing the sibling of the patient’s disease despite privacy concerns is... |
| GT10 | Supporting genetic testing despite privacy concerns is.... |
| GT11 | For insurers, requesting genetic tests from healthy adults in order to assess their health risks is ... |
| GT12 | Using public health funds on expensive gene therapies is ... |
| GT13 | Taking into account the genetic profile of applicants with respect to genetic diseases when hiring a kindergarten teacher is... |
| GT14 | Mitigating a criminal sentence due to the offender’s genetic predisposition is ... |
| GT15 | Using genome editing on consenting adults to enhance their cognitive performance is ... |
| GT16 | Changing the genomes of human embryos for medical research without destroying them is... |
| GT17 | Changing the genomes of human embryos to ensure they will not develop a fatal disease is ... |
| GT18 | Genome editing of human adults to protect them against influenza is ... |
| GT19 | Changing the genome of human embryos to ensure they will not get influenza is ... |
| GT20 | Using risky genome editing therapies for the medical treatment of cancer patients is ... |
| GT21 | Testing for the risk of genome editing on consenting adults is ... |
| GT22 | Using genome editing to enhance the cognitive development of human embryos in underprivileged families is ... |
| GT23 | Changing the genome of farm animals in order to improve their wellbeing is ... |
| GT24 | Editing the genome of farm animals to reduce costs without harming them is ... |
| GT25 | Editing the genome of crops in order to fight world poverty is ... |
| GT26 | Editing the genome of foods to improve their taste is ... |
| GT27 | Editing the genome of animals to make it possible for animal organs to be transplanted to humans is ... |
| GT28 | Editing the genome of crops to improve their nutritional value is... |
| GT29 | Editing the genome of wild animals to make them immune against certain diseases is ... |
| GT30 | Editing the genome of plants to improve crops for farming is... |

*Note.* Answers were given on a 6-point Likert scale ranging from (1) “morally bad” to (6) “morally good”. Italicized items are included in the GTQ20, bold items are included in the GTQ5.
